# Ethyl *N*-[2-(4-phen­oxy­phen­oxy)eth­yl]carbamate

**DOI:** 10.1107/S1600536812037105

**Published:** 2012-09-05

**Authors:** Jolanta Karpinska, Manuel Kuhs, Ake Rasmuson, Andrea Erxleben, Patrick McArdle

**Affiliations:** a School of Chemistry, National University of Ireland, Galway, University road, Galway, Ireland; b School of Chemistry, Limerick, Ireland

## Abstract

The title compound, C_17_H_19_NO_4_, which is a non-toxic insect growth regulator with the common name fenoxycarb, contains two independent and conformationally different mol­ecules in the asymmetric unit. Although the inter-ring dihedral angles are similar [62.21 (15) and 63.00 (14)°], the side-chain orientations differ. In the crystal, the mol­ecules are linked through N—H⋯O hydrogen-bonding associations, giving chains which extend along [110], while intra- and inter­molecular aromatic C—H⋯π inter­actions give sheet structures parallel to [110].

## Related literature

For studies on the role of fenoxycarb as an insect growth regulator, see: Paya *et al.* (2009 ▶); Sullivan (2010 ▶); Kavallieratos *et al.* (2012 ▶); Goncu & Parlak (2012 ▶). For related structures containing the phenyl ether motif, see: Ammon *et al.* (1983 ▶); Clayden *et al.* (1990 ▶); Glidewell *et al.* (2005 ▶). For hydrogen-bond data, see Lifson *et al.* (1979 ▶).
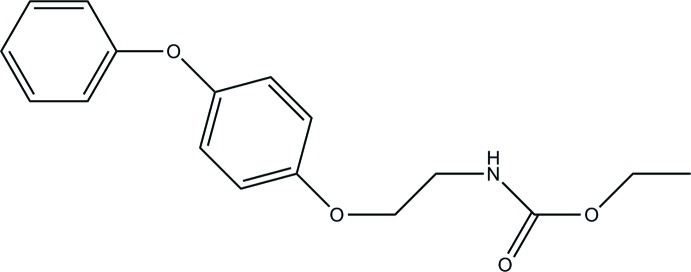


## Experimental

### 

#### Crystal data

C_17_H_19_NO_4_
*M* *_r_* = 301.33
Triclinic, 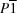

*a* = 5.9035 (3) Å
*b* = 7.5712 (4) Å
*c* = 35.373 (2) Å
α = 85.618 (5)°
β = 86.213 (5)°
γ = 89.046 (4)°
*V* = 1572.88 (15) Å^3^
*Z* = 4
Mo *K*α radiation
μ = 0.09 mm^−1^
*T* = 150 K
0.40 × 0.30 × 0.15 mm

#### Data collection

Oxford Diffraction Xcalibur, Sapphire3 CCD-detector diffractometer
Absorption correction: multi-scan (*CrysAlis PRO*; Oxford Diffraction, 2010 ▶) *T*_min_ = 0.894, *T*_max_ = 1.000
25379 measured reflections
5745 independent reflections
2937 reflections with *I* > 2σ(*I*)
*R*_int_ = 0.074

#### Refinement

*R*[*F* ^2^ > 2σ(*F* ^2^)] = 0.063
*wR*(*F* ^2^) = 0.126
*S* = 0.97
5745 reflections
399 parameters
H-atom parameters constrained
Δρ_max_ = 0.21 e Å^−3^
Δρ_min_ = −0.22 e Å^−3^

### 

Data collection: *CrysAlis PRO* (Oxford Diffraction, 2010 ▶); cell refinement: *CrysAlis PRO*; data reduction: *CrysAlis PRO*; program(s) used to solve structure: *SHELXS97* (Sheldrick, 2008 ▶); program(s) used to refine structure: *SHELXL97* (Sheldrick, 2008 ▶); molecular graphics: *SORTX* (McArdle, 1995 ▶); software used to prepare material for publication: *SHELXL97*.

## Supplementary Material

Crystal structure: contains datablock(s) global, I. DOI: 10.1107/S1600536812037105/zs2230sup1.cif

Structure factors: contains datablock(s) I. DOI: 10.1107/S1600536812037105/zs2230Isup2.hkl

Supplementary material file. DOI: 10.1107/S1600536812037105/zs2230Isup3.cml

Additional supplementary materials:  crystallographic information; 3D view; checkCIF report

## Figures and Tables

**Table 1:** Hydrogen-bond geometry (Å, °) *Cg*1, *Cg*2, *Cg*3 and *Cg*4 are the centroids of the C1–C6, C7–C12, C18–C23 and C24–C29 rings, respectively.

*D*—H⋯*A*	*D*—H	H⋯*A*	*D*⋯*A*	*D*—H⋯*A*
N1—H1*N*1⋯O7^i^	0.86	1.96	2.815 (4)	178
N2—H2*N*2⋯O3	0.86	1.94	2.795 (4)	173
C2—H2⋯*Cg*3^ii^	0.93	2.85	3.562 (3)	134
C5—H5⋯*Cg*3^iii^	0.93	2.85	3.562 (3)	134
C9—H9⋯*Cg*4	0.93	2.86	3.622 (3)	139
C12—H12⋯*Cg*4^i^	0.93	2.84	3.590 (3)	138
C19—H19⋯*Cg*1	0.93	2.92	3.657 (3)	137
C22—H22⋯*Cg*1^iv^	0.93	2.84	3.583 (3)	137
C26—H26⋯*Cg*2^v^	0.93	2.87	3.579 (3)	134
C29—H29⋯*Cg*2^vi^	0.93	2.80	3.527 (3)	135

Symmetry codes: (i) 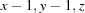; (ii) 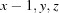; (iii) 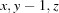; (iv) 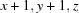; (v) 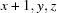; (vi) 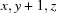.

**Fig. 1.**
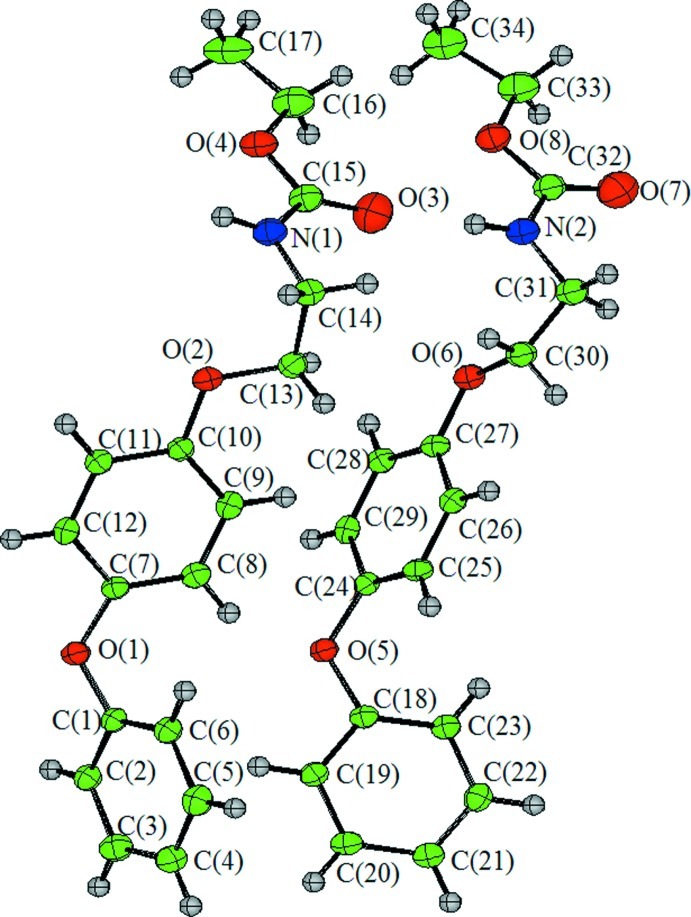
The atom numbering scheme for the two independent molecules in the asymmetric unit of fenoxycarb. Displacement ellipsoids for non-H atoms are are drawn at the 30% probability level.

**Fig. 2.**
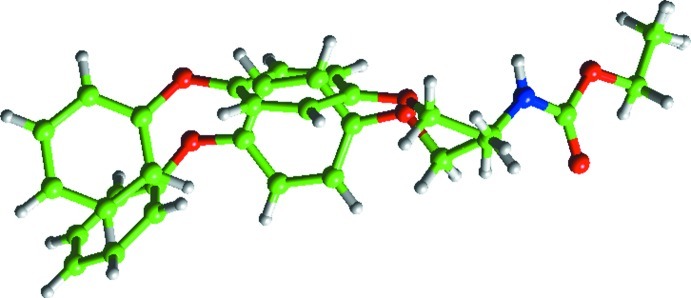
Molecular overlay of the two conformationally different fenoxycarb molecules.

**Fig. 3.**
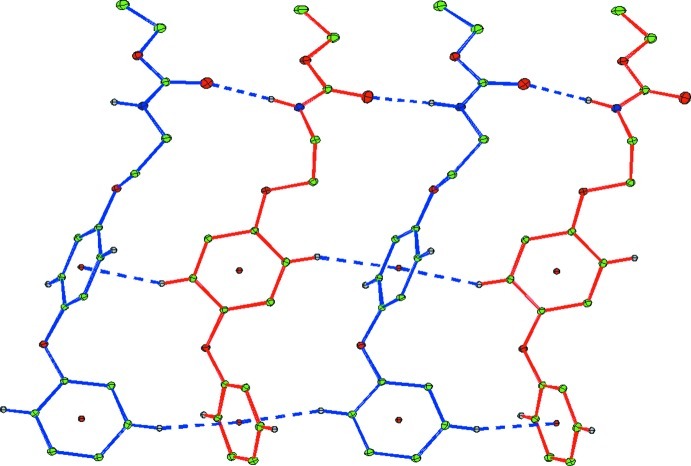
The one-dimensional hydrogen-bonded chains in the crystal structure of fenoxycarb.

**Fig. 4.**
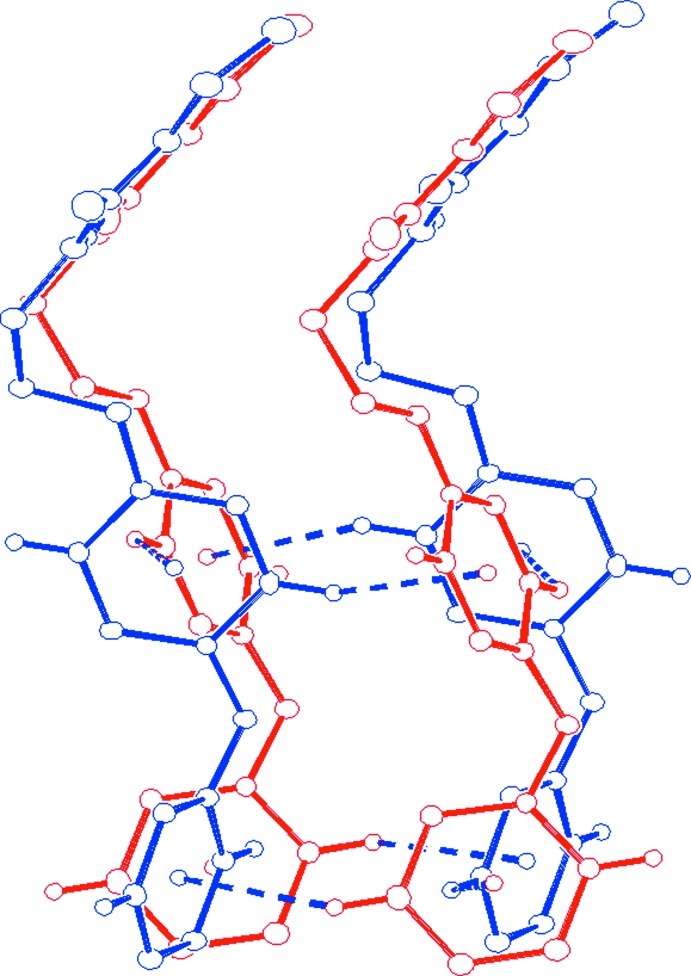
A view down the H-bonded chains showing C—H···π interactions.

**Fig. 5.**
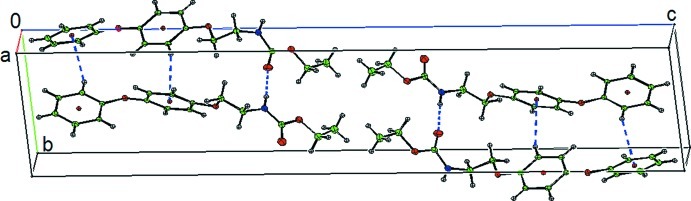
The unit cell packing of fenoxycarb viewed down a.

## supplementary crystallographic information

### Comment

The title compound fenoxycarb, C_17_H_19_NO_4_ is an insect growth regulator with juvenile hormone activity (Paya *et al.*, 2009; Sullivan *et al.*, 2010; Kavallieratos *et al.*, 2012; Goncu & Parlak, 2012). As part of our studies on the crystallization behaviour of this compound the crystal structure of fenocarb has been determined.

There are two chemically identical but conformationally different molecules in the asymmetric unit (Fig. 1). Although the two benzene rings adopt similar orientations [inter-ring dihedral angles, 62.21 (15) and 63.00 (14)°], the side chains differ in the region associated with the intermolecular N—H···O hydrogen-bonding interactions between the two molecules, with torsion angles O2—C13—C14—N1 and C13—C14—N1—C15 [-64.6 (4) and -85.2 (4)° in molecule 1] and O6—C30—C31—N2 and C30—C31—N2—C32 [57.4 (3) and -151.2 (3)° in molecule 2] (Fig. 2). The N2—H···O3 and N1—H···O3^i^ hydrogen bonds bonds (Table 1) generate a one-dimensional motif that is parallel to [110] (Fig. 3). The hydrogen bonds are estimated to be of moderate strength at 8 kcal/mol (Lifson *et al.*, 1979) and they are supported by weaker C—H···π contacts. With the aromatic ring centroids numbered in order of carbon number the distances for the interactions H26···*Cg2*, H29···*Cg2*, H9···*Cg4* and H12···*Cg4* are 2.87, 2.80, 2.86 and 2.86 Å respectively. The one-dimensional chains are built into two-dimensional sheets by further C—H···π contacts involving H19···*Cg1*, H22···*Cg1*, H2···*Cg3* and H5···*Cg3* are 2.92, 2.84, 2.85 and 2.85 Å respectively (Fig. 4). There are no π-π contacts involving the aromatic rings as adjacent rings are non-coplanar. The unit cell packing is shown in Fig.5.

### Experimental

The title compound of high purity (>98.8%) was obtained from Syngenta, Switzerland. Colourless, lath shaped crystals were grown from a solution in ethanol by slow evaporation at room temperature.

### Refinement

All H atoms were included in the refinement in calculated positions [N—H = 0.88 Å, C—H(aromatic) = 0.93 Å, C—H(methylene) = 0.97 Å or C—H(methyl) = 0.96 Å] and allowed to ride on their parent atoms, with *U*_iso_(H) = 1.2*U*_eq_ (C, N) or 1.5*U*_eq_(C methyl).

### Crystal data

**Table:**

C_17_H_19_NO_4_	*Z* = 4
*M**_r_* = 301.33	*F*(000) = 640
Triclinic, *P*1	*D*_x_ = 1.273 Mg m^−^^3^
Hall symbol: -P 1	Mo *K*α radiation, λ = 0.7107 Å
*a* = 5.9035 (3) Å	Cell parameters from 2643 reflections
*b* = 7.5712 (4) Å	θ = 2.8–29.2°
*c* = 35.373 (2) Å	µ = 0.09 mm^−^^1^
α = 85.618 (5)°	*T* = 150 K
β = 86.213 (5)°	Parallelepiped, colourless
γ = 89.046 (4)°	0.40 × 0.30 × 0.15 mm
*V* = 1572.88 (15) Å^3^	

### Data collection

**Table:**

Oxford Diffraction Xcalibur, Sapphire3 CCD-detector diffractometer	5745 independent reflections
Radiation source: Enhance (Mo) X-ray Source	2937 reflections with *I* > 2σ(*I*)
Graphite monochromator	*R*_int_ = 0.074
Detector resolution: 16.1048 pixels mm^-1^	θ_max_ = 25.4°, θ_min_ = 2.8°
ω scans	*h* = −6→7
Absorption correction: multi-scan (*CrysAlis PRO*; Oxford Diffraction, 2010)	*k* = −9→9
*T*_min_ = 0.894, *T*_max_ = 1.000	*l* = −42→36
25379 measured reflections	

### Refinement

**Table:**

Refinement on *F*^2^	Primary atom site location: structure-invariant direct methods
Least-squares matrix: full	Secondary atom site location: difference Fourier map
*R*[*F*^2^ > 2σ(*F*^2^)] = 0.063	Hydrogen site location: inferred from neighbouring sites
*wR*(*F*^2^) = 0.126	H-atom parameters constrained
*S* = 0.97	*w* = 1/[σ^2^(*F*_o_^2^) + (0.0202*P*)^2^] where *P* = (*F*_o_^2^ + 2*F*_c_^2^)/3
5745 reflections	(Δ/σ)_max_ < 0.001
399 parameters	Δρ_max_ = 0.21 e Å^−^^3^
0 restraints	Δρ_min_ = −0.22 e Å^−^^3^

### Special details

**Table:**

Geometry. All e.s.d.'s (except the e.s.d. in the dihedral angle between two l.s. planes) are estimated using the full covariance matrix. The cell e.s.d.'s are taken into account individually in the estimation of e.s.d.'s in distances, angles and torsion angles; correlations between e.s.d.'s in cell parameters are only used when they are defined by crystal symmetry. An approximate (isotropic) treatment of cell e.s.d.'s is used for estimating e.s.d.'s involving l.s. planes.
Refinement. Refinement of *F*^2^ against ALL reflections. The weighted *R*-factor *wR* and goodness of fit *S* are based on *F*^2^, conventional *R*-factors *R* are based on *F*, with *F* set to zero for negative *F*^2^. The threshold expression of *F*^2^ > σ(*F*^2^) is used only for calculating *R*-factors(gt) *etc*. and is not relevant to the choice of reflections for refinement. *R*-factors based on *F*^2^ are statistically about twice as large as those based on *F*, and *R*- factors based on ALL data will be even larger.

### Fractional atomic coordinates and isotropic or equivalent isotropic displacement parameters (Å^2^)

**Table:**

	*x*	*y*	*z*	*U*_iso_*/*U*_eq_	
O1	−0.0999 (3)	0.0094 (3)	0.14727 (6)	0.0292 (6)	
O2	0.2377 (3)	−0.0627 (3)	0.28990 (6)	0.0284 (6)	
O4	0.2198 (4)	0.1319 (3)	0.41170 (6)	0.0397 (7)	
N1	0.3603 (5)	−0.0324 (4)	0.36637 (8)	0.0341 (7)	
H1N1	0.2510	−0.1030	0.3739	0.044*	
O3	0.5364 (4)	0.2199 (3)	0.37656 (8)	0.0543 (8)	
C1	0.0310 (5)	0.0155 (4)	0.11300 (9)	0.0229 (8)	
C2	−0.0716 (5)	0.1035 (4)	0.08305 (9)	0.0260 (8)	
H2	−0.2109	0.1606	0.0871	0.034*	
C3	0.0335 (6)	0.1065 (4)	0.04699 (10)	0.0329 (9)	
H3	−0.0349	0.1651	0.0266	0.043*	
C4	0.2414 (6)	0.0216 (4)	0.04128 (10)	0.0330 (9)	
H4	0.3129	0.0235	0.0170	0.043*	
C5	0.3429 (6)	−0.0661 (4)	0.07175 (10)	0.0336 (9)	
H5	0.4828	−0.1224	0.0679	0.044*	
C6	0.2356 (5)	−0.0698 (4)	0.10793 (9)	0.0266 (8)	
H6	0.3019	−0.1295	0.1284	0.035*	
C7	0.0047 (5)	−0.0086 (4)	0.18145 (9)	0.0222 (8)	
C8	0.1958 (5)	0.0809 (4)	0.18832 (9)	0.0268 (8)	
H8	0.2701	0.1513	0.1688	0.035*	
C9	0.2801 (5)	0.0672 (4)	0.22452 (9)	0.0270 (8)	
H9	0.4104	0.1276	0.2290	0.035*	
C10	0.1681 (5)	−0.0371 (4)	0.25359 (9)	0.0239 (8)	
C11	−0.0258 (5)	−0.1266 (4)	0.24658 (9)	0.0241 (8)	
H11	−0.1013	−0.1966	0.2660	0.031*	
C12	−0.1071 (5)	−0.1122 (4)	0.21074 (9)	0.0246 (8)	
H12	−0.2376	−0.1722	0.2062	0.032*	
C13	0.4498 (5)	0.0105 (4)	0.29752 (9)	0.0306 (9)	
H13A	0.4353	0.1378	0.2987	0.040*	
H13B	0.5647	−0.0137	0.2776	0.040*	
C14	0.5151 (5)	−0.0751 (4)	0.33510 (9)	0.0339 (9)	
H14A	0.5202	−0.2026	0.3338	0.044*	
H14B	0.6662	−0.0372	0.3398	0.044*	
C15	0.3848 (6)	0.1153 (5)	0.38376 (10)	0.0326 (9)	
C16	0.2336 (6)	0.2855 (5)	0.43345 (11)	0.0469 (11)	
H16A	0.2367	0.3934	0.4168	0.061*	
H16B	0.3700	0.2795	0.4474	0.061*	
C17	0.0265 (7)	0.2828 (5)	0.46030 (10)	0.0620 (13)	
H17A	−0.1068	0.2913	0.4461	0.087*	
H17B	0.0295	0.3811	0.4758	0.087*	
H17C	0.0239	0.1741	0.4762	0.087*	
O5	0.4018 (3)	0.4830 (3)	0.14990 (6)	0.0274 (6)	
O6	0.7396 (3)	0.4935 (3)	0.29179 (6)	0.0293 (6)	
O8	0.6720 (4)	0.6213 (3)	0.41569 (7)	0.0368 (6)	
N2	0.8499 (4)	0.4950 (4)	0.36737 (8)	0.0337 (7)	
H2N2	0.7451	0.4168	0.3709	0.044*	
O7	0.9987 (4)	0.7375 (3)	0.38924 (7)	0.0562 (8)	
C18	0.5324 (5)	0.5007 (4)	0.11550 (9)	0.0236 (8)	
C19	0.4372 (5)	0.4303 (4)	0.08561 (9)	0.0256 (8)	
H19	0.3003	0.3704	0.0894	0.033*	
C20	0.5461 (5)	0.4492 (4)	0.05006 (9)	0.0285 (8)	
H20	0.4841	0.3999	0.0298	0.037*	
C21	0.7470 (5)	0.5407 (4)	0.04410 (9)	0.0310 (9)	
H21	0.8186	0.5553	0.0199	0.040*	
C22	0.8410 (5)	0.6104 (4)	0.07428 (9)	0.0296 (9)	
H22	0.9779	0.6702	0.0704	0.039*	
C23	0.7322 (5)	0.5919 (4)	0.11049 (9)	0.0243 (8)	
H23	0.7938	0.6404	0.1308	0.032*	
C24	0.5056 (5)	0.4846 (4)	0.18424 (9)	0.0218 (8)	
C25	0.6998 (5)	0.3895 (4)	0.19176 (9)	0.0251 (8)	
H25	0.7755	0.3277	0.1729	0.033*	
C26	0.7829 (5)	0.3860 (4)	0.22775 (9)	0.0241 (8)	
H26	0.9151	0.3227	0.2329	0.031*	
C27	0.6686 (5)	0.4770 (4)	0.25594 (9)	0.0226 (8)	
C28	0.4693 (5)	0.5692 (4)	0.24816 (9)	0.0252 (8)	
H28	0.3905	0.6286	0.2671	0.033*	
C29	0.3880 (5)	0.5730 (4)	0.21237 (9)	0.0240 (8)	
H29	0.2548	0.6348	0.2072	0.031*	
C30	0.9338 (5)	0.3936 (4)	0.30377 (9)	0.0307 (9)	
H30A	1.0497	0.3917	0.2830	0.040*	
H30B	0.8916	0.2726	0.3118	0.040*	
C31	1.0216 (5)	0.4823 (5)	0.33638 (9)	0.0366 (9)	
H31A	1.1511	0.4156	0.3456	0.048*	
H31B	1.0726	0.6003	0.3275	0.048*	
C32	0.8522 (6)	0.6256 (5)	0.39035 (10)	0.0343 (9)	
C33	0.6632 (6)	0.7599 (5)	0.44188 (10)	0.0467 (11)	
H33A	0.6760	0.8752	0.4280	0.061*	
H33B	0.7870	0.7452	0.4586	0.061*	
C34	0.4383 (6)	0.7456 (5)	0.46465 (10)	0.0549 (12)	
H34A	0.3173	0.7534	0.4477	0.077*	
H34B	0.4230	0.8402	0.4813	0.077*	
H34C	0.4314	0.6339	0.4795	0.077*	

### Atomic displacement parameters (Å^2^)

**Table:**

	*U*^11^	*U*^22^	*U*^33^	*U*^12^	*U*^13^	*U*^23^
O1	0.0267 (13)	0.0378 (15)	0.0230 (14)	0.0023 (11)	−0.0034 (10)	−0.0012 (11)
O2	0.0311 (14)	0.0302 (14)	0.0238 (14)	−0.0025 (11)	−0.0039 (10)	0.0008 (11)
O4	0.0506 (17)	0.0392 (16)	0.0299 (16)	−0.0070 (13)	0.0029 (13)	−0.0109 (12)
N1	0.0404 (19)	0.0306 (18)	0.0310 (19)	−0.0116 (15)	0.0027 (14)	−0.0018 (14)
O3	0.0543 (18)	0.0398 (17)	0.069 (2)	−0.0240 (15)	0.0118 (15)	−0.0133 (15)
C1	0.0230 (19)	0.0206 (18)	0.025 (2)	0.0017 (15)	−0.0006 (15)	−0.0030 (15)
C2	0.026 (2)	0.0239 (19)	0.028 (2)	0.0029 (16)	−0.0054 (15)	−0.0003 (16)
C3	0.040 (2)	0.028 (2)	0.031 (2)	−0.0004 (18)	−0.0102 (17)	0.0003 (17)
C4	0.035 (2)	0.034 (2)	0.030 (2)	−0.0045 (18)	0.0020 (17)	−0.0077 (17)
C5	0.032 (2)	0.030 (2)	0.040 (2)	0.0057 (17)	−0.0042 (18)	−0.0065 (18)
C6	0.027 (2)	0.0239 (19)	0.029 (2)	0.0033 (16)	−0.0048 (16)	−0.0033 (16)
C7	0.027 (2)	0.0185 (18)	0.0217 (19)	0.0059 (15)	−0.0040 (15)	−0.0054 (15)
C8	0.031 (2)	0.0200 (19)	0.028 (2)	0.0001 (16)	0.0019 (15)	0.0026 (16)
C9	0.027 (2)	0.0198 (19)	0.033 (2)	0.0002 (16)	−0.0024 (16)	0.0009 (16)
C10	0.025 (2)	0.0218 (19)	0.024 (2)	0.0077 (16)	−0.0020 (15)	−0.0018 (15)
C11	0.027 (2)	0.0189 (18)	0.026 (2)	0.0015 (15)	0.0045 (15)	−0.0020 (15)
C12	0.0228 (19)	0.0198 (19)	0.031 (2)	0.0023 (15)	0.0022 (15)	−0.0040 (16)
C13	0.028 (2)	0.036 (2)	0.028 (2)	−0.0001 (17)	−0.0012 (15)	−0.0051 (17)
C14	0.037 (2)	0.036 (2)	0.029 (2)	0.0015 (18)	−0.0076 (17)	−0.0024 (18)
C15	0.040 (2)	0.026 (2)	0.032 (2)	−0.0031 (19)	−0.0043 (18)	0.0004 (17)
C16	0.065 (3)	0.037 (2)	0.041 (3)	0.005 (2)	−0.012 (2)	−0.014 (2)
C17	0.088 (4)	0.064 (3)	0.033 (3)	0.016 (3)	0.001 (2)	−0.008 (2)
O5	0.0264 (13)	0.0333 (14)	0.0226 (14)	−0.0003 (11)	−0.0027 (10)	−0.0021 (11)
O6	0.0344 (14)	0.0284 (14)	0.0259 (14)	0.0044 (11)	−0.0061 (10)	−0.0040 (11)
O8	0.0417 (16)	0.0343 (15)	0.0355 (16)	−0.0025 (13)	−0.0021 (12)	−0.0097 (12)
N2	0.0393 (19)	0.0333 (18)	0.0290 (19)	−0.0151 (15)	−0.0020 (14)	−0.0027 (15)
O7	0.0579 (18)	0.0566 (19)	0.056 (2)	−0.0336 (16)	0.0018 (14)	−0.0151 (15)
C18	0.029 (2)	0.0190 (19)	0.022 (2)	0.0051 (16)	−0.0019 (15)	0.0034 (15)
C19	0.027 (2)	0.0247 (19)	0.025 (2)	−0.0003 (16)	−0.0047 (15)	0.0028 (16)
C20	0.037 (2)	0.027 (2)	0.023 (2)	0.0020 (17)	−0.0100 (16)	−0.0037 (16)
C21	0.035 (2)	0.030 (2)	0.026 (2)	0.0056 (18)	0.0002 (16)	0.0062 (17)
C22	0.027 (2)	0.027 (2)	0.034 (2)	0.0011 (16)	0.0000 (16)	0.0015 (17)
C23	0.031 (2)	0.0177 (18)	0.025 (2)	0.0017 (16)	−0.0052 (15)	−0.0056 (15)
C24	0.0215 (19)	0.0222 (18)	0.0219 (19)	−0.0054 (15)	−0.0032 (15)	−0.0008 (15)
C25	0.033 (2)	0.0215 (19)	0.0206 (19)	0.0036 (16)	0.0019 (15)	−0.0045 (15)
C26	0.026 (2)	0.0194 (18)	0.027 (2)	0.0048 (15)	−0.0021 (15)	−0.0029 (15)
C27	0.031 (2)	0.0164 (18)	0.0206 (19)	−0.0041 (15)	−0.0032 (15)	0.0014 (14)
C28	0.028 (2)	0.0211 (19)	0.026 (2)	0.0004 (16)	0.0031 (15)	−0.0048 (15)
C29	0.0223 (19)	0.0155 (18)	0.034 (2)	0.0015 (15)	−0.0011 (15)	−0.0030 (15)
C30	0.033 (2)	0.035 (2)	0.025 (2)	0.0031 (17)	−0.0075 (16)	0.0015 (17)
C31	0.035 (2)	0.046 (2)	0.029 (2)	−0.0075 (19)	−0.0038 (17)	−0.0005 (18)
C32	0.035 (2)	0.039 (2)	0.028 (2)	−0.005 (2)	−0.0076 (18)	0.0056 (18)
C33	0.061 (3)	0.043 (3)	0.040 (3)	0.003 (2)	−0.013 (2)	−0.018 (2)
C34	0.066 (3)	0.056 (3)	0.044 (3)	0.014 (2)	−0.005 (2)	−0.011 (2)

### Geometric parameters (Å, º)

**Table:**

O1—C7	1.390 (3)	O5—C18	1.396 (3)
O1—C1	1.392 (3)	O5—C24	1.397 (3)
O2—C10	1.373 (3)	O6—C27	1.377 (3)
O2—C13	1.428 (3)	O6—C30	1.432 (3)
O4—C15	1.352 (4)	O8—C32	1.344 (4)
O4—C16	1.449 (4)	O8—C33	1.450 (4)
N1—C15	1.331 (4)	N2—C32	1.328 (4)
N1—C14	1.440 (4)	N2—C31	1.450 (4)
N1—H1N1	0.8600	N2—H2N2	0.8600
O3—C15	1.206 (4)	O7—C32	1.218 (4)
C1—C6	1.368 (4)	C18—C23	1.372 (4)
C1—C2	1.377 (4)	C18—C19	1.376 (4)
C2—C3	1.380 (4)	C19—C20	1.373 (4)
C2—H2	0.9300	C19—H19	0.9300
C3—C4	1.386 (4)	C20—C21	1.380 (4)
C3—H3	0.9300	C20—H20	0.9300
C4—C5	1.387 (4)	C21—C22	1.378 (4)
C4—H4	0.9300	C21—H21	0.9300
C5—C6	1.389 (4)	C22—C23	1.393 (4)
C5—H5	0.9300	C22—H22	0.9300
C6—H6	0.9300	C23—H23	0.9300
C7—C8	1.370 (4)	C24—C25	1.374 (4)
C7—C12	1.388 (4)	C24—C29	1.384 (4)
C8—C9	1.400 (4)	C25—C26	1.392 (4)
C8—H8	0.9300	C25—H25	0.9300
C9—C10	1.386 (4)	C26—C27	1.387 (4)
C9—H9	0.9300	C26—H26	0.9300
C10—C11	1.386 (4)	C27—C28	1.391 (4)
C11—C12	1.381 (4)	C28—C29	1.381 (4)
C11—H11	0.9300	C28—H28	0.9300
C12—H12	0.9300	C29—H29	0.9300
C13—C14	1.504 (4)	C30—C31	1.503 (4)
C13—H13A	0.9700	C30—H30A	0.9700
C13—H13B	0.9700	C30—H30B	0.9700
C14—H14A	0.9700	C31—H31A	0.9700
C14—H14B	0.9700	C31—H31B	0.9700
C16—C17	1.497 (5)	C33—C34	1.508 (5)
C16—H16A	0.9700	C33—H33A	0.9700
C16—H16B	0.9700	C33—H33B	0.9700
C17—H17A	0.9600	C34—H34A	0.9600
C17—H17B	0.9600	C34—H34B	0.9600
C17—H17C	0.9600	C34—H34C	0.9600
			
C7—O1—C1	120.0 (2)	C18—O5—C24	120.2 (2)
C10—O2—C13	117.9 (2)	C27—O6—C30	119.1 (2)
C15—O4—C16	116.0 (3)	C32—O8—C33	115.3 (3)
C15—N1—C14	120.6 (3)	C32—N2—C31	121.0 (3)
C15—N1—H1N1	119.7	C32—N2—H2N2	119.5
C14—N1—H1N1	119.7	C31—N2—H2N2	119.5
C6—C1—C2	121.6 (3)	C23—C18—C19	121.5 (3)
C6—C1—O1	124.1 (3)	C23—C18—O5	123.6 (3)
C2—C1—O1	114.1 (3)	C19—C18—O5	114.8 (3)
C1—C2—C3	119.5 (3)	C20—C19—C18	119.4 (3)
C1—C2—H2	120.2	C20—C19—H19	120.3
C3—C2—H2	120.2	C18—C19—H19	120.3
C2—C3—C4	119.8 (3)	C19—C20—C21	120.5 (3)
C2—C3—H3	120.1	C19—C20—H20	119.8
C4—C3—H3	120.1	C21—C20—H20	119.8
C3—C4—C5	120.0 (3)	C22—C21—C20	119.6 (3)
C3—C4—H4	120.0	C22—C21—H21	120.2
C5—C4—H4	120.0	C20—C21—H21	120.2
C4—C5—C6	120.0 (3)	C21—C22—C23	120.4 (3)
C4—C5—H5	120.0	C21—C22—H22	119.8
C6—C5—H5	120.0	C23—C22—H22	119.8
C1—C6—C5	119.0 (3)	C18—C23—C22	118.6 (3)
C1—C6—H6	120.5	C18—C23—H23	120.7
C5—C6—H6	120.5	C22—C23—H23	120.7
C8—C7—C12	119.6 (3)	C25—C24—C29	120.7 (3)
C8—C7—O1	123.9 (3)	C25—C24—O5	123.0 (3)
C12—C7—O1	116.2 (3)	C29—C24—O5	116.0 (3)
C7—C8—C9	120.5 (3)	C24—C25—C26	119.7 (3)
C7—C8—H8	119.7	C24—C25—H25	120.1
C9—C8—H8	119.7	C26—C25—H25	120.1
C10—C9—C8	119.5 (3)	C27—C26—C25	120.0 (3)
C10—C9—H9	120.2	C27—C26—H26	120.0
C8—C9—H9	120.2	C25—C26—H26	120.0
O2—C10—C11	115.7 (3)	O6—C27—C26	125.9 (3)
O2—C10—C9	124.5 (3)	O6—C27—C28	114.5 (3)
C11—C10—C9	119.8 (3)	C26—C27—C28	119.6 (3)
C12—C11—C10	120.1 (3)	C29—C28—C27	120.2 (3)
C12—C11—H11	119.9	C29—C28—H28	119.9
C10—C11—H11	119.9	C27—C28—H28	119.9
C11—C12—C7	120.4 (3)	C28—C29—C24	119.8 (3)
C11—C12—H12	119.8	C28—C29—H29	120.1
C7—C12—H12	119.8	C24—C29—H29	120.1
O2—C13—C14	107.1 (3)	O6—C30—C31	107.3 (3)
O2—C13—H13A	110.3	O6—C30—H30A	110.3
C14—C13—H13A	110.3	C31—C30—H30A	110.3
O2—C13—H13B	110.3	O6—C30—H30B	110.3
C14—C13—H13B	110.3	C31—C30—H30B	110.3
H13A—C13—H13B	108.6	H30A—C30—H30B	108.5
N1—C14—C13	113.0 (3)	N2—C31—C30	112.0 (3)
N1—C14—H14A	109.0	N2—C31—H31A	109.2
C13—C14—H14A	109.0	C30—C31—H31A	109.2
N1—C14—H14B	109.0	N2—C31—H31B	109.2
C13—C14—H14B	109.0	C30—C31—H31B	109.2
H14A—C14—H14B	107.8	H31A—C31—H31B	107.9
O3—C15—N1	125.2 (4)	O7—C32—N2	125.0 (4)
O3—C15—O4	123.6 (3)	O7—C32—O8	122.8 (4)
N1—C15—O4	111.1 (3)	N2—C32—O8	112.2 (3)
O4—C16—C17	106.3 (3)	O8—C33—C34	107.3 (3)
O4—C16—H16A	110.5	O8—C33—H33A	110.2
C17—C16—H16A	110.5	C34—C33—H33A	110.2
O4—C16—H16B	110.5	O8—C33—H33B	110.2
C17—C16—H16B	110.5	C34—C33—H33B	110.2
H16A—C16—H16B	108.7	H33A—C33—H33B	108.5
C16—C17—H17A	109.5	C33—C34—H34A	109.5
C16—C17—H17B	109.5	C33—C34—H34B	109.5
H17A—C17—H17B	109.5	H34A—C34—H34B	109.5
C16—C17—H17C	109.5	C33—C34—H34C	109.5
H17A—C17—H17C	109.5	H34A—C34—H34C	109.5
H17B—C17—H17C	109.5	H34B—C34—H34C	109.5
			
C7—O1—C1—C6	30.9 (4)	C24—O5—C18—C23	−29.0 (4)
C7—O1—C1—C2	−154.4 (3)	C24—O5—C18—C19	155.6 (3)
C6—C1—C2—C3	−0.1 (5)	C23—C18—C19—C20	1.0 (5)
O1—C1—C2—C3	−174.9 (3)	O5—C18—C19—C20	176.5 (3)
C1—C2—C3—C4	−0.3 (5)	C18—C19—C20—C21	−1.2 (5)
C2—C3—C4—C5	0.1 (5)	C19—C20—C21—C22	1.3 (5)
C3—C4—C5—C6	0.4 (5)	C20—C21—C22—C23	−1.3 (5)
C2—C1—C6—C5	0.6 (5)	C19—C18—C23—C22	−0.9 (4)
O1—C1—C6—C5	174.9 (3)	O5—C18—C23—C22	−176.0 (3)
C4—C5—C6—C1	−0.7 (5)	C21—C22—C23—C18	1.0 (5)
C1—O1—C7—C8	42.6 (4)	C18—O5—C24—C25	−45.6 (4)
C1—O1—C7—C12	−143.3 (3)	C18—O5—C24—C29	141.2 (3)
C12—C7—C8—C9	0.6 (5)	C29—C24—C25—C26	−1.8 (5)
O1—C7—C8—C9	174.5 (3)	O5—C24—C25—C26	−174.7 (3)
C7—C8—C9—C10	−0.4 (5)	C24—C25—C26—C27	0.6 (5)
C13—O2—C10—C11	173.2 (3)	C30—O6—C27—C26	−7.7 (4)
C13—O2—C10—C9	−5.6 (4)	C30—O6—C27—C28	175.5 (3)
C8—C9—C10—O2	178.8 (3)	C25—C26—C27—O6	−175.7 (3)
C8—C9—C10—C11	0.0 (4)	C25—C26—C27—C28	0.9 (5)
O2—C10—C11—C12	−178.8 (3)	O6—C27—C28—C29	175.8 (3)
C9—C10—C11—C12	0.0 (5)	C26—C27—C28—C29	−1.2 (4)
C10—C11—C12—C7	0.2 (5)	C27—C28—C29—C24	0.0 (5)
C8—C7—C12—C11	−0.6 (5)	C25—C24—C29—C28	1.5 (5)
O1—C7—C12—C11	−174.9 (3)	O5—C24—C29—C28	174.9 (3)
C10—O2—C13—C14	−165.2 (3)	C27—O6—C30—C31	160.0 (3)
C15—N1—C14—C13	−85.2 (4)	C32—N2—C31—C30	−151.2 (3)
O2—C13—C14—N1	−64.6 (4)	O6—C30—C31—N2	57.3 (4)
C14—N1—C15—O3	−3.0 (5)	C31—N2—C32—O7	−4.1 (5)
C14—N1—C15—O4	178.8 (3)	C31—N2—C32—O8	176.0 (3)
C16—O4—C15—O3	−0.2 (5)	C33—O8—C32—O7	0.0 (5)
C16—O4—C15—N1	178.1 (3)	C33—O8—C32—N2	179.9 (3)
C15—O4—C16—C17	175.8 (3)	C32—O8—C33—C34	173.6 (3)

### Hydrogen-bond geometry (Å, º)

Cg1, Cg2, Cg3 and Cg4 are the centroids of the C1–C6, C7–C12, C18–C23 and C24–C29 rings,respectively.

**Table:**

*D*—H···*A*	*D*—H	H···*A*	*D*···*A*	*D*—H···*A*
N1—H1*N*1···O7^i^	0.86	1.96	2.815 (4)	178
N2—H2*N*2···O3	0.86	1.94	2.795 (4)	173
C2—H2···*Cg*3^ii^	0.93	2.85	3.562 (3)	134
C5—H5···*Cg*3^iii^	0.93	2.85	3.562 (3)	134
C9—H9···*Cg*4	0.93	2.86	3.622 (3)	139
C12—H12···*Cg*4^i^	0.93	2.84	3.590 (3)	138
C19—H19···*Cg*1	0.93	2.92	3.657 (3)	137
C22—H22···*Cg*1^iv^	0.93	2.84	3.583 (3)	137
C26—H26···*Cg*2^v^	0.93	2.87	3.579 (3)	134
C29—H29···*Cg*2^vi^	0.93	2.80	3.527 (3)	135

Symmetry codes: (i) *x*−1, *y*−1, *z*; (ii) *x*−1, *y*, *z*; (iii) *x*, *y*−1, *z*; (iv) *x*+1, *y*+1, *z*; (v) *x*+1, *y*, *z*; (vi) *x*, *y*+1, *z*.

## References

[bb1] Ammon, H. L., Bhattacharjee, S. K., Ravi, P. S. & Potlock, S. J. (1983). *Acta Cryst.* C**39**, 304–306.

[bb2] Clayden, N. J., Williams, D. & O’Mahoney, C. A. (1990). *J. Chem. Soc. Perkin Trans. 2*, pp. 729–733.

[bb3] Glidewell, C., Low, J. N., Skakle, J. M. S. & Wardell, J. L. (2005). *Acta Cryst.* C**61**, o185–o187. 10.1107/S0108270105002076 15750251

[bb4] Goncu, E. & Parlak, O. (2012). *Fol. Histochem. Cytobiol. Pol. Acad. Sci. Pol. Histochem. Cytochem. Soc.* **50**, 52–57. 10.2478/18696 22532136

[bb5] Kavallieratos, N. G., Athanassiou, C. G., Vayias, B. J. & Tomanovic, Z. (2012). *J. Food Prot.* **75**, 942–950. 10.4315/0362-028X.JFP-11-397 22564945

[bb6] Lifson, S., Hagler, A. T. & Dauber, P. (1979). *J. Am. Chem. Soc.* **101**, 5111–5121.

[bb7] McArdle, P. (1995). *J. Appl. Cryst.* **28**, 65. 10.1107/S1600576721008529 PMC8493623 34667454

[bb8] Oxford Diffraction (2010). *CrysAlis PRO* Oxford Diffraction Ltd, Yarnton, England.

[bb9] Paya, P., Oliva, J., Zafrilla, P., Camara, M. A. & Barba, A. (2009). *Ecotoxicology*, **18**, 1137–1142. 10.1007/s10646-009-0394-2 19636704

[bb10] Sheldrick, G. M. (2008). *Acta Cryst.* A**64**, 112–122. 10.1107/S0108767307043930 18156677

[bb11] Sullivan, J. J. (2010). *Reviews of Environmental Contamination and Toxicology*, Vol 202, edited by D. M. Whitacre, pp. 155–184. New York: Springer. 10.1007/978-1-4419-1157-5_3 19898762

